# CortexVR: Immersive analysis and training of cognitive executive functions of soccer players using virtual reality and machine learning

**DOI:** 10.3389/fpsyg.2022.754732

**Published:** 2022-08-23

**Authors:** Christian Krupitzer, Jens Naber, Jan-Philipp Stauffert, Jan Mayer, Jan Spielmann, Paul Ehmann, Noel Boci, Maurice Bürkle, André Ho, Clemens Komorek, Felix Heinickel, Samuel Kounev, Christian Becker, Marc Erich Latoschik

**Affiliations:** ^1^Department Food Informatics and Computational Science Lab, Universität Hohenheim, Stuttgart, Germany; ^2^Chair of Information Systems II, Universtiät Mannheim, Mannheim, Germany; ^3^HCI Group, Universität Würzburg, Würzburg, Germany; ^4^TSG Research Lab, Zuzenhaussen, Germany; ^5^Software Enngineering Group, Universität Würzburg, Würzburg, Germany

**Keywords:** sports analytics, virtual worlds training simulations, training of cognitive functions, executive functions, healthcare, machine learning, training

## Abstract

**Goal:**

This paper presents an immersive Virtual Reality (VR) system to analyze and train Executive Functions (EFs) of soccer players. EFs are important cognitive functions for athletes. They are a relevant quality that distinguishes amateurs from professionals.

**Method:**

The system is based on immersive technology, hence, the user interacts naturally and experiences a training session in a virtual world. The proposed system has a modular design supporting the extension of various so-called game modes. Game modes combine selected game mechanics with specific simulation content to target particular training aspects. The system architecture decouples selection/parameterization and analysis of training sessions *via* a coaching app from an Unity3D-based VR simulation core. Monitoring of user performance and progress is recorded by a database that sends the necessary feedback to the coaching app for analysis.

**Results:**

The system is tested for VR-critical performance criteria to reveal the usefulness of a new interaction paradigm in the cognitive training and analysis of EFs. Subjective ratings for overall usability show that the design as VR application enhances the user experience compared to a traditional desktop app; whereas the new, unfamiliar interaction paradigm does not negatively impact the effort for using the application.

**Conclusion:**

The system can provide immersive training of EF in a fully virtual environment, eliminating potential distraction. It further provides an easy-to-use analyzes tool to compare user but also an automatic, adaptive training mode.

## 1. Introduction

Principle performance characteristics of many professional sport activities are continuously evolving, for example, professional soccer is characterized by shorter ball possession times, increased need of accurate passing rates, or longer running distances. The average duration of ball possession per contact for soccer players decreased from 2.8 s in 2006 to 1.1 s in 2010 (Carling, 2010). Consequently, players need to train to react faster. However, today's high training load with repeated physical training of athletic skills in combination with an ever increased number of matches in various competition contexts already puts significant physical strain on players. Hence, professional clubs are seeking for alternatives. A promising approach focuses on non-physical training of so-called Executive Functions (EFs) to increase the players' *Soccer IQ* (Ingle, 2016). EFs encompass the cognitive abilities to evaluate and make situational decisions (Strauss et al., 2006). Hence, they are mental determinants of players' behavior and can be an important discriminant for comparing players as well as determining suitable positions for youth players as different positions have different requirements regarding the EFs (Verburgh et al., 2014).

EFs can effectively be increased by certain computer games (Green et al., 2012; Vestberg et al., 2012; Verburgh et al., 2014) which already led to an adoption of similar approaches by professional sports. One of the most elaborated systems, the Helix, is based on Mixed Reality (MR). Here, soccer players stand in the focal center of an 180° large screen display to partly immerse them into a virtual soccer game. During training, players have to execute a multi-object tracking task in form of tracking non-player characters (NPCs) running around on the virtual soccer field. Due to the large size of the room-filling screen, this tasks demands high capabilities in working memory, peripheral vision, and cognitive flexibility. Recently, the Helix has been extend toward a 360° screen-based systems; however, the mentioned shortcomings are still present.

However, the current MR-based Helix has several shortcomings. First, it's NPC logic is very rudimentary and based on random paths. As a consequence, users with a good understanding of the game do not benefit from that. Second, the player has to tell a coach which NPCs should be selected. Thus, the reaction time of a player is not measurable, hence, a detailed analysis of the player's performance taking the trade-off between accuracy and reaction time into account is not feasible. Third, while the Helix already allows a semi-immersion for players based on the large-screen installation, it restricts analysis and training to one user at a time, it is a complex and expensive system, and does not scale well with large teams. The semi-immersiveness of the current approach is potentially disadvantageous. While a high degree of immersion does not necessarily have to be a goal for any VR system (Bowman and McMahan, 2007), it is usually beneficial when it comes to the elicitation of a strong effect of a place illusion and spatial presence. The semi-immersiveness also risks to have players notice the physical space around them, e.g., when the visual attention is directed to the vicinity of screen borders.

This paper presents *CortexVR*, an immersive VR system for the analysis and training of EFs of soccer players^1^. We present an immersive system that aims at eliminating the need of a large screen system that suffers the issue of distraction while providing a whole new type of user interaction and training experience. Bird (2020) showed the applicability of using virtual reality head-mounted displays within applied sport psychology. Accordingly, our system is based on a head-mounted display (HMD) VR kit (the *Oculus Rift*) that effectively shuts out visual and auditory distractors from the physical space around users. This avoids breaks in presence and fosters undisturbed training of EFs. The system platform increases mobility, lowers hardware costs, and allows an increased number of users concurrently training. *CortexVR* incorporates automated assessment of reaction times. The system's modular design fosters extensibility and includes necessary control functions for training personal *via* a dedicated app. The current version includes realistic NPC paths captured from a real professional soccer match in the German Bundesliga. Additionally, we offer a game mode which dynamically adjusts the game level depending on the performance of the user. The system is tested for VR-critical performance criteria and evaluated for the ease of use and user experience. Subjective ratings for overall usability show that the user experience created by using VR increases the user experience while analysis/training of EFs. Additionally, we evaluate the reinforcement learning approach of the adaptive game mode.

The remainder of this paper is structured as follows. Section 2 discusses related work: the relevant theoretical fundamentals of EFs, works in the field of training EFs, and the application of VR in sports. Subsequently, Section 3 covers the analysis of the requirements for the platform based on an analysis of the Helix and stakeholder interviews. Within Section 4, we describe the technology stack of the *CortexVR* platform as well as the adaptive game mode based on reinforcement learning. Section 5 describes the qualitative evaluation with a group of users and the quantitative of the adaptive game mode, followed by the discussion of the results in Section 6. Section 7 concludes this paper.

## 2. Related work

This section introduces the theoretical foundations of EFs. Following, it presents different approaches for training EFs as well as VR-based sports training in general. Lastly, we discuss the related work and motivate the research gap for this work.

### 2.1. Executive functions

Executive Functions (EFs) denote cognitive processes controlling human actions in different environments (Strauss et al., 2006). EFs usually are separated into two distinct types: The work here focuses on basic cognitive processes including cognitive inhibition, working memory, and cognitive flexibility. The higher order EFs (e.g., reasoning and/or problem solving) are not considered.

Cognitive inhibition refers to the blocking out or tuning out of information that is irrelevant to the task at hand (Harnishfeger, 1995). This mental process can be intentional or unintentional and can manifest itself in a variety of ways (Harnishfeger, 1995). The working memory is a conceptualization of human memory of limited capacity responsible for storage and manipulation of information over brief temporal intervals (Baddeley, 2010). Cognitive flexibility refers to the ability to adapt to the transition from thinking and reasoning about one concept to a different one (Scott, 1962).

EFs develop gradually across one's lifespan. They can be improved at any point in life. Neuropsychological tests, as Stroop test and rating scales, are used to measure EFs. Notably, there were several approaches to use VR for the assessment of EF for various use cases (see, e.g., Pugnetti et al., 1998; Lalonde et al., 2013; Climent-Martinez et al., 2014). EFs are considered to have a high relevance for sports. In several experiments soccer players performed better than non-athletes concerning their EFs, especially in decision making tasks (Vestberg et al., 2012; Verburgh et al., 2014). Also for other sports, the training of EFs seems to be promising (Holfelder et al., 2020; Koch and Krenn, 2021). De Waelle et al. (2021) identified that the strength of EFs is higher for team sports player than for self-paced sports and that those effects are already present for young athletes.

### 2.2. Training of executive functions

#### 2.2.1. VR-based training

Since EFs are highly relevant for the success of a professional soccer player, it is beneficial to train these functions to increase player performance. However, this is not restricted to high performance sports. Kubesch and Walk (2009) discuss the effects of training EFs for children. The training of these functions is often used in treatment of several, especially cognitive, diseases, such as Parkinson's disease (Sammer et al., 2006) or children with Attention Deficit Hyperactivity Disorder (ADHD) (Tamm et al., 2014). It has extensively been explored for neurological deficits in the area of neurorehabilitation (McGeorge et al., 2001; Lo Priore et al., 2003; Weiss et al., 2006). The analysis of the EFs is neglected in all those approaches.

#### 2.2.2. Gamification-based training

Studies showed that action video games can be used to train specific EFs, e.g., gain advantages in task-switching (Green et al., 2012), increase the processing speed (Dye et al., 2009), or enhance the development of perceptual templates (Bejjanki et al., 2014). While these studies show that video games have a positive training effect, they are rarely used systematically to train the performance of professional athletes.

### 2.3. VR-based sports training

Akbaş et al. (2019) provide an overview of the application of VR for competitive sports. They identified three categories of applications: performance analysis, simulation improvement, and virtual training.

First approaches of VR-based sports training included simple videos as VR lessons (Success Series, 2019) for amateur sports or advanced systems with elaborated special-purpose training equipment aimed at gyms (Icaros, 2019). But VR is not only relevant for the private sports sector; it is also discovered in professional sports for training and analysis. Bideau et al. (2004, 2010) analyzed and trained the movement of handball goal keepers with synthetic VR opponents. Additionally, there are first commercial products for VR sports training emerging. *NeuroTrainer* aims at training neurological and cognitive functions of athletes (NeuroTrainer, 2019). A more physical approach is the *Virtual Goalie* by Reaction VR Sports which makes use of the motion tracking controllers of the HTC Vive and Oculus Rift to train the reaction of lacrosse goalies (Reaction VR Sports, 2019). *Mi Hiepa Sports* introduced a mixed reality approach for soccer training: motion tracking sensors are attached to different parts of the player's body and his movement is fully captured in the virtual environment (Mi Hiepa Sports, 2019). This can be used to train technical skills by studying a 360° perspective. *StriVR* provides a VR tool to train different plays in a high repetition without the physical demand (StriVR, 2019). This tool is dominantly used in American Football to train play calls. A further example is EonSports VR (EonSports VR, 2019), which is mainly used in baseball for technical and tactical training. Besides professional sports training, VR helped to enable exercises of groups in a remotely fashion during the COVID-19 pandemic. For example, Gao et al. (2020) present a study on exercises for health and wellness of older adults during the COVID-19 Pandemic.

### 2.4. Distinction from related work

The main advantage of VR used for sports analysis and training is the possibility to achieve a high number of repetitions and a good memorization effect without the physical stress of conventional athletics training, as could be illustrated by various applications. Further, as the technology disappears, a new, pervasive user experience is generated which supports the training effects as it avoids distraction. Therefore, it is especially useful during physiotherapy and regeneration, but also as a low-impact add-on to conventional physical training. VR-based training of EFs has shown to be effective in neurorehabilitation (McGeorge et al., 2001; Lo Priore et al., 2003; Weiss et al., 2006) but the current state-of-the-art in professional soccer still relies on gamified approaches. To the best of our knowledge, there currently is no research available which uses VR to analyze/train EFs and applies this to professional soccer as is illustrated by the approach in this paper. Hence, this paper provides the first approach that transfers the analysis/training of EFs in an immersive VR system—which has been proven to be beneficial in neurorehabilitation—into the domain of sports, namely into professional soccer.

## 3. Requirements analysis

**Figure 1:**
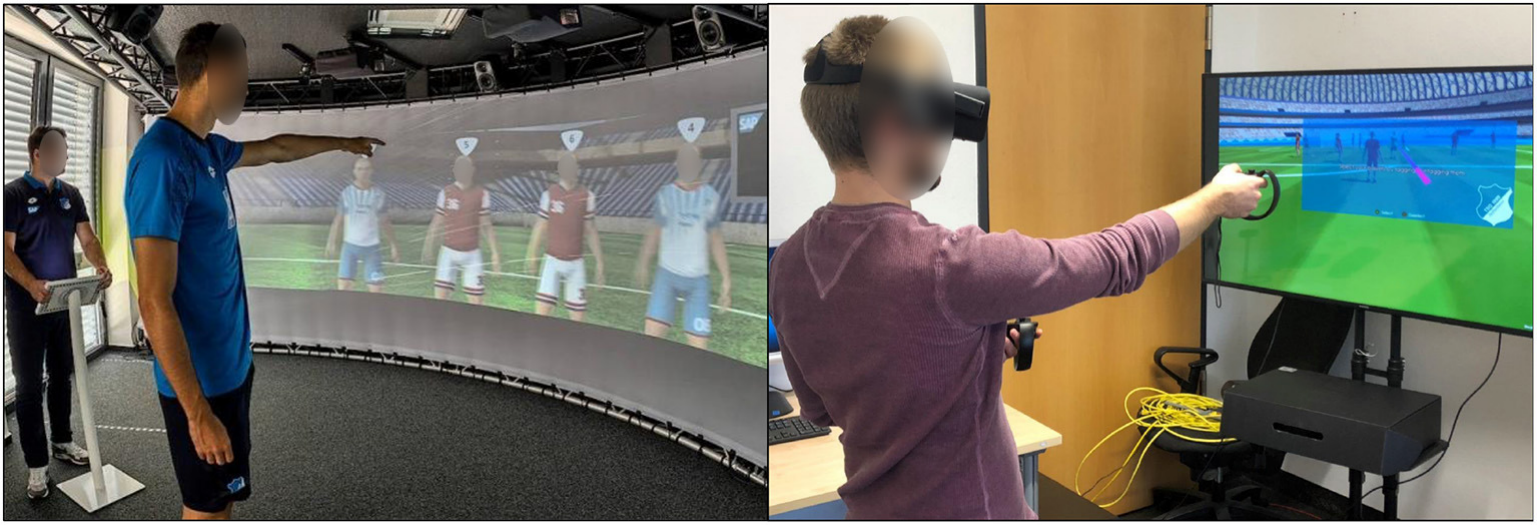
The left part of the figure shows the original *Helix* for training of EFs on a 180° screen. It is a room-filling 180° screen (source: Michael Horeni, 2017). The right part of the figure shows the new *CortexVR* for VR-supported training of EFs with an Oculus Rift used by one of the authors.

The soccer club TSG Hoffenheim uses the Helix to analyze and train the EFs of professional first league soccer players—both male and female—as well as the youth players. The players have to track NPCs in a simulated game environment. The Helix currently comprises an 180° screen by integrating the stream of six different projectors in a wide-angle screen. A recent extension of the system provides a 360° screen. The Helix app integrates two roles: the coach and the user, i.e., the soccer players that train their EFs. The coach uses a tablet to configure the game settings before starting the game. The user is then confronted with a randomly created sequence of soccer NPCs running on the field. Their task is to tell the coach which NPCs were highlighted in the beginning of the sequence. The coach then selects these NPCs in the application. Figure 1 shows on the left the original Helix system with both roles, coach and user, simultaneously active during the selection of the NPCs^2^. We did an analysis of the limitations of the original Helix system and conducted interviews with stakeholders. Based on this material and the analysis of related approaches, we derived several requirements for a computing system to support immersive cognitive training of EFs for professional soccer players. In the following, we describe these functional requirements.

First, an important requirement is cross-platform support. Using VR, still requires setup and maintenance effort. As the necessary IT skills cannot be presupposed in a soccer club, we decided to design the application in a way that it can be used as immersive VR application but also as normal desktop application with mouse/key support (requirement *R*_*App*_1). This way, soccer clubs can study the benefits of immersion for training of EFs but have a backup option in case that the organizational structures cannot guarantee the use of VR devices. However, we suppose that immersion has benefits for training EFs compared to the normal desktop application.

The coach should be able to configure games (requirement *R*_*Config*_1), setup the training session (requirement *R*_*Config*_2), and analyze the player's performance (requirement *R*_*Config*_3). As this functionality neither affects the training itself nor should be running as a VR app, the configuration application should be a stand-alone tool (requirement *R*_*Config*_4).

One limitation in the original Helix system is that the coach enters the data collected verbally from the user, resulting in a delay while data input. Hence, the reaction time of a soccer player is not trackable. To overcome this, the user should be able to directly interact with the application (requirement *R*_*App*_2) through a controller or gesture-based input.

Soccer players with a good anticipation of the game should benefit from that while tracking. Consequently, we decided to include the option to choose between player movements extracted from real spatiotemporal soccer tracking data or random paths (requirement *R*_*App*_3.1). Furthermore, it should be possible to use specific game situations like corner kicks or kick-offs extracted from real soccer game data (requirement *R*_*App*_3.2). The reproducibility of those sequences—in contrast to the random paths of the original Helix system—helps to systematically analyze and compare the EFs of players.

In addition to the configuration of the game itself, the *CortexVR* application should also offer new game modes to enhance the gameplay and make it more diversified (requirement *R*_*App*_4). The user therefore is not bound to the repetition of the same game but can find challenges in new variants which triggers the gamification aspects. Thus, it is important to strive for modularity (requirement *R*_*App*_5)—such as offering modules for (i) generating the game situations, (ii) rendering the game situations, or (iii) evaluating user data—as several functionalities are relevant across game modes. Further, the system needs to follow a design that allows to extend the system with additional game modes (requirement *R*_*App*_6).

## 4. Design and implementation

**Figure 2:**
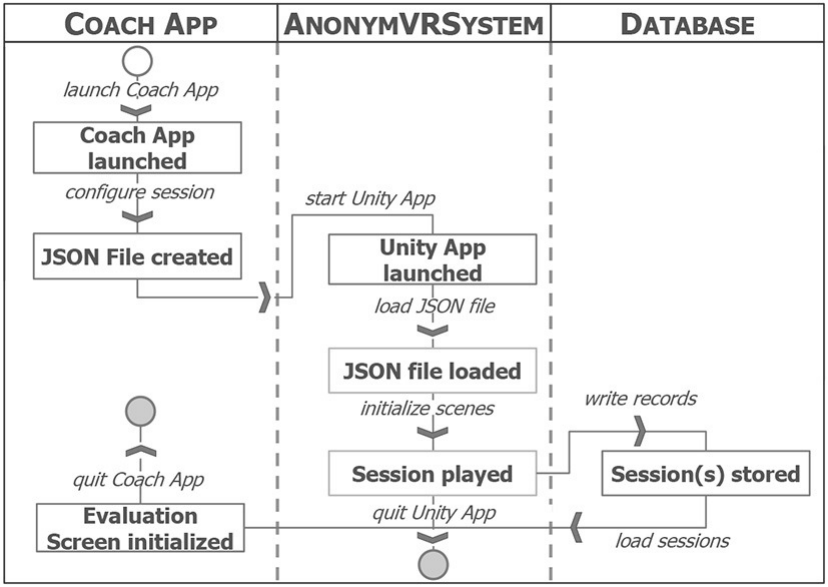
Interaction between the *Coach App* and the *CortexVR* application.

As the requirements *R*_*Config*_1–*R*_*Config*_3 demand that game configuration and evaluation are running in a dedicated app (see requirement *R*_*Config*_4), it was decided to implement those in the so called *Coach App*. Figure 2 visualizes the interaction between *Coach App* and *CortexVR*. By launching the *Coach App*, the instructor—i.e., a coach or staff member—starts the system. Then, the session configuration takes place through which the instructor decides on the game parameters and stores them to a respective JSON file. In the next step, after starting the Unity3D App and loading the JSON file, the scene is initialized. The original Helix application generates random paths for the movement of NPCs. For generating realistic player movement paths (see requirement *R*_*App*_3.1) and extracting clearly defined game situations (see requirement *R*_*App*_3.2), we use the spatiotemporal data of a German Bundesliga soccer game. It was recorded by the widely-used tracking system *TRACAB Image Tracking* and provides two-dimensional positions of all players and three-dimensional position of the ball. After playing the configured numbers of sessions, the Unity3D App writes the game log data into a shared SQLite database before it is closed. The instructor can then use the *Coach App* for analyzing the player's performance. In the following, we explain the design and implementation of the *CortexVR* and the *Coach App*. Afterwards, we describe the design of the three game modes.

### 4.1. Coach App

The *Coach App* enables the management of user and team data, the initialization of training sessions as well as the analysis of user performance. It is a standalone .NET based application implemented in C# using the Windows Presentation Foundation (WPF). Using the UI, the coach can define the training session for a soccer player (see requirement *R*_*Config*_1). This includes the definition of the game mode and the level. This way, the coach can define sessions for analysis and comparison of players before starting the app (see requirement *R*_*Config*_2).

The *Coach App* integrates an overview for the analysis of a player's performance (see requirement *R*_*Config*_3). The game produces session data that captures the user's performance in terms of accuracy and reaction time. Also, the comparison between the performance of the user under consideration and a certain peer group is offered by the *Coach App*. The peer group as well as all parameters of the training sessions can be filtered to ensure a customized analysis.

### 4.2. Cortex VR

The *CortexVR* app displays the content of the training session and interacts with the user. It loads the session configuration, starts the game mode, and stores the data for the evaluation of the user's performance. We implemented the *CortexVR* app using the Unity3D Game Engine. Unity3D enables cross-compilation for various platforms including VR devices (see requirement *R*_*App*_1).

All game modes use the same control procedure and the same UI. Accordingly, these elements are modularized which fulfills requirement *R*_*App*_5. We designed the games modes after discussions with staff and coaches of the soccer club TSG Hoffenheim. For fulfilling the requirement of direct interaction between the app and the user (see requirement *R*_*App*_2), we decided to use the *Oculus Rift* VR device as it includes—besides a headset—two gesture-based controllers and a set of sensors called Constellation system which is responsible for the tracking of the position of the user's head as well as other VR devices. However, it would be possible to migrate our *CortexVR* application to the Occulus Go and avoid the necessity of having a dedicated base station machine.

**Figure 3:**
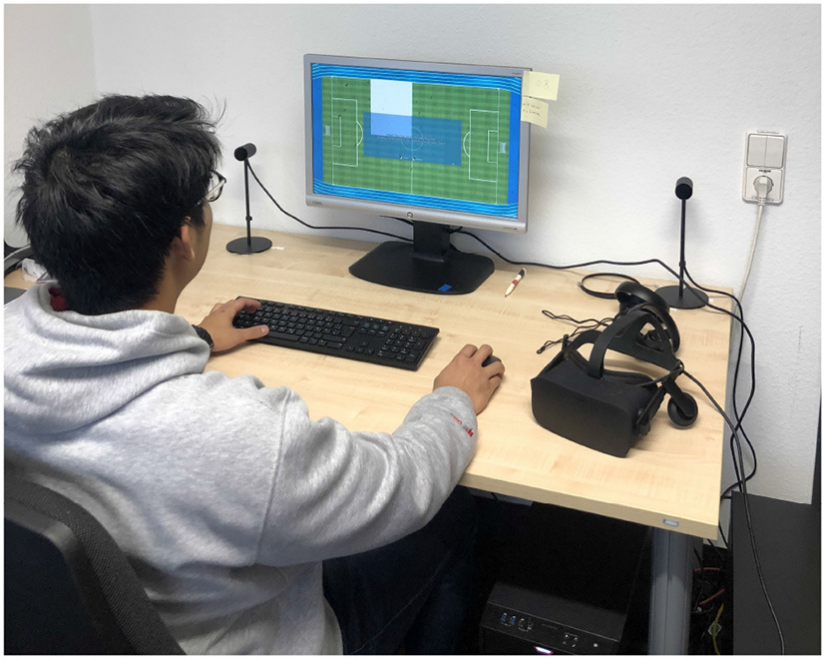
Setup of the evaluation for the mouse/key control-based application used by one of the authors.

We implemented two variants of the application: a low immersive desktop-based version and a high immersive VR version using the Oculus Rift. Both offer the same set of functionality. However, they differ in immersiveness and user interaction. The desktop version relies on mouse and keyboard input (see Figure 3), the VR version uses the input of the controllers and sensors to display a laser beam like pointer for enabling a natural interaction in a virtual environment.

For triggering the gamification effect, we created a realistic environment for the user. As stadium we used an asset from the Unity3D Asset Store. A sound game object that holds a mp3 with stadium sounds in the audio source component simulates a real stadium atmosphere. We created two versions of a NPC using *Adobe Fuse CC*: one with a blue T-shirt and black shorts; the other with a red T-shirt and red shorts. All other visual parameters are the same, including their body specifications and facial features, to increase the level of complexity.

### 4.3. Game modes

The original Helix system targeted the training of the working memory as one specific sub-function of EFs. However, it only provides a single game mode for this specific purpose. We converted the tracking player mode to our new *CortexVR* system. Supported by our modularized approach (see requirement *R*_*App*_5) and as new game modes extend a common abstract class (see requirement *R*_*App*_6), it is really convenient to add new game modes to the system by re-using several existing modules. As divergent learning experiences in sports can enhance training of the cognitive executive functions (Buning et al., 2021), we add two additional game modes (see requirement *R*_*App*_4). In the following, we present the design of the three implemented game modes.

#### 4.3.1. Tracking players

The *Tracking Players* game mode consists of a fair number of NPCs being targeted at the beginning. Then, the user tries to follow them and pointing them out at the end of the game session. The key difference to the original Helix is that the user can now move within the environment and manipulate the game camera which as a result means that he is not restricted to a frontal view only anymore. Accordingly, we shift the game environment from a 180° screen to a more realistic 360° screen. This widens the field of view, but also offers a new challenge due to the possibility of NPCs being off screen. Hence, the use of the working memory is stressed additionally, as the user has to remember the different positions of the NPCs as well as which of them had been marked in the beginning.

#### 4.3.2. Count players

The *Count Players* game mode aims to improve the EFs of the users by letting them keep track of the count of current NPCs on the screen. As NPCs can appear from the left and right, but also from behind, the eyes are trained to keep the overview on fixed positions of the display instead of following specific objects as in the Tracking Players game mode. This game mode addresses the training of the working memory but also of the inhibition, as the user spontaneously has to decide if a player has been counted already. The camera is fixed which means that moving and looking around is disabled. To vary the difficulty, the position can be adjusted, e.g., inside or near the NPCs but also bird-like views that simplify the task. This game mode does not require soccer knowledge because the user does not need to interpret the movement of NPCs.

#### 4.3.3. Find ball

In the *Find Ball* mode, the user sees a game situation without the ball and needs to estimate the position of the ball. Therefore, the soccer pitch is split into equally sized areas. The user needs to analyze the overall formation and position of the NPCs. The game situations are extracted from real game data, accordingly, soccer-related knowledge helps to analyze the game situation. However, animations like shooting and passing are not available which raises the difficulty. This game mode supports the training of cognitive flexibility, as it targets the ability of players to transfer their game understanding to the unknown situations that miss important information, such as the ball and animations of players' actions.

#### 4.3.4. Reinforcement learning-supported adaptive game mode

The additional *track adaptive* game mode extends the *tracking players* mode and offers the functionality of adapting the speed of the application depending on the performance of the user. Accordingly, this increases the difficulty quickly and supports an improved analysis as players are higher demanded. We decided to rely on reinforcement learning (RL) as this machine learning technique is often applied in artificial game intelligence [e.g., in Backgammon (Tesauro, 1995), Hearts (Sturtevant and White, 2007), or first-person shooters (McPartland and Gallagher, 2011)]. We applied Q-learning for predicting the attribute Speed of NPCs using the current state and a list of possible actions. The objective of Q-learning is to learn the quality of actions (policy) for telling an agent which action to perform based on the expected rewards. As state, we consider the speed, the amount of correctly played rounds, the accuracy, and the time. The speed is split into 10 areas, reflecting velocities between 13 and 40 $\frac{\text{km}}{\text{h}}$ (in steps of 3 $\frac{\text{km}}{\text{h}}$). Successful rounds are those in which the user has tracked all NPCs correctly. As we always track two players, the accuracy is 0, 0.5, or 1 for each round. For the time, we assume that users requires at least 1.5 s and ignore all values above 30 s.


(1) $$T(n)=\{\begin{array}{l} \lfloor \text{SubmitTime}/3\rceil -1\text{ if }n\leq \text{30}\\ \;9\text{ if }n>\text{30} \end{array}$$

As actions, we focus on adjusting the velocity of NPCs by increasing or reducing the velocity in multiples of 3 $\frac{\text{km}}{\text{h}}$. To keep the speed always between 13 and 40 $\frac{\text{km}}{\text{h}}$, only specific actions are possible. For example, when starting with 13 $\frac{\text{km}}{\text{h}}$, increases can be arbitrarily (9 actions) or the speed stays the same (1 action). For determining the quality of an action, positive or negative rewards are assigned. Our reward function takes into account if the player already played more than one correct round and the correctly played rounds on the current level. Further, it distinguishes if the speed has recently decreased or increased.

## 5. Evaluation

This section presents the evaluation of our approach for an immersive system to support the analysis and training of EFs. The evaluation is composed of an analysis of important technical properties, a user study as well as a quantitative analysis of the adaptive game mode's RL method. In the following, we describe in consecutive order the evaluation.

### 5.1. Technical evaluation of *CortexVR*

To guarantee the performance of the VR mode, we conducted motion to photon latency measurements with frame counting.

#### 5.1.1. Methodology

A camera recorded both the physical controller and the screen at 240 frames per second. The latency is the time between the start of a movement of the physical controller until the start of the movement of its virtual counterpart. The ideal case of recording both the Oculus Rift screen and the physical controller is not possible as the lenses render the image unusable. The measurement is conducted in two steps: The motion to photon latency is determined by observing the real controller in front of and the virtual controller on the computer's monitor screen. The reaction time difference between the monitor screen and the Oculus Rift screen is determined by observing how fast they react to color changes spanning the entire image. Frames per second are derived with Unity's unscaledDeltaTime. It indicates for each frame how much time has passed since the previous frame. The technical measures are taken on a desktop PC with Intel Core i7 7,700 k 4 × 4.2 GHz, 16 GB RAM, and Nvidia Geforce GTX 1080.

#### 5.1.2. Results

The measured latency between controller movement and its virtual counterpart on the monitor is 66.6 ms (SD = 21.6 ms). The difference between the monitor screen and the Oculus Rift screen is 32.2 ms (SD = 5.8 ms). This leads to a motion to photon latency between the physical controller and the Oculus Rift screen of 34.4 ms (SD = 27.4ms). The application performed at a mean of 78.39 frames per second, i.e., a mean frame time of 12.75 ms (SD = 3.96 ms). Accordingly, the performance of the VR presentation is fast enough so that the technical implementation of the VR mode does not negatively influence the user experience (UX).

### 5.2. User study

For the evaluation of our approach, we focus the analysis of the UX in a virtual world using the VR-based solution in contrast to the traditional desktop app. As we expect that most users did not have experience in VR applications before, we assume that those might expect a higher effort in using the VR app due to initial familiarization. Accordingly, we want to discuss the effort expected by users for a new interaction type. Especially, we want to guarantee that the analysis of EFs does not suffer from less experience in using the VR technology. However, we still expect that the integration of VR improves the gamification and, hence, the UX. Accordingly, we investigate the following two hypotheses:

- *H1: VR has increased expected effort for users and, hence, affect negatively the analysis of EFs*.
- *H2: VR enhances the Hedonic Motivation and, hence, affect positively the analysis of EFs*.

#### 5.2.1. Procedure

Each participant of the user study had to play the three game modes in the following specified order: (i) *Track Players* game, (ii) *Count Players* game, and (iii) *Find Ball* game. For each game mode, the participants were asked to play it four times to eliminate learning effects using both types of control, VR and non-VR control. The order of the two control types was chosen randomly with a toss coin for each participant at the beginning, but was equal for all three game modes for the user. Throughout the evaluation, the participants were supported by our staff for questions. Further, we provided a short introduction. Afterwards, participants answered a questionnaire.

#### 5.2.2. Setup

We used the Oculus Rift VR device for the graphics output. The participants could interact with the application using the included Touch controllers and sensors. The configuration of the VR device and the controllers were done beforehand by our staff. As base station, we used a desktop PC with Intel Core i7 8,700 k 6 × 3.7 GHz, 32 GB RAM, and Nvidia Geforce GTX 1080Ti running with Windows 10 and Unity3D 2018.2.13. The right part of Figure 1 illustrates the environment with the Oculus Rift. Except of the control and the fact, that the content is shown on a regular display, the non-VR variant (see Figure 3) is identical to the VR one.

#### 5.2.3. Methodology

We focus on the two dimensions of Effort Expectancy (cf. hypothesis *H1*) and Hedonic Motivation (cf. hypothesis *H2*). Effort Expectancy describes “the degree of ease for using the system the user expects” (Venkatesh et al., 2003). We expect that users might be unfamiliar with VR applications, hence, have a higher effort for using it. We operationalized the construct Effort Expectancy for each game mode using the question items provided by Venkatesh et al. (2003) regarding the ease of use and learnability. However, we expect that VR apps will provide a better user experience. Hedonic motivation describes “the fun or pleasure derived from using a technology” (Venkatesh et al., 2012). It showed to play an important role in determining technology acceptance and use, especially in gamification settings as the *CortexVR* application. We conceptualized Hedonic Motivation using question items from Venkatesh et al. (2012) and one additional item regarding the game atmosphere. Accordingly, we evaluate the trade-off of user effort for using (unfamiliar) VR and the benefits of a better analysis of EFs through a more realistic UX. Table 1 shows the question items for the user study. The question items are operationalized with 5-points Likert scales from 1 (strongly disagree) to 5 (strongly agree) and the option to skip question items. Except of the question item *HC*_4_, we asked the same questions for both versions of the *CortexVR*–the one with VR control and the non-VR version with mouse/key control—and for each game mode.

**Table 1:** Constructs of the user study (GameModeX stands for the (i) Track, (ii) Count, or (iii) Find Ball game modes).

**ID**	**Question items for the user study**
	*Effort expectancy* (Venkatesh et al., 2003)
*EE*_1_	Learning how to use GameModeX with VR/mouse is easy for me.
*EE*_2_	My interaction with the GameModeX with VR/mouse is clear and understandable.
*EE*_3_	I find the GameModeX with VR/mouse easy to use.
*EE*_4_	It is easy for me to become skillful at using the GameModeX with VR/mouse.
	*Hedonic motivation* (Venkatesh et al., 2012)
*HC*_1_	Playing the GameModeX with VR/mouse is fun.
*HC*_2_	Playing the GameModeX with VR/mouse is enjoyable.
*HC*_3_	Playing the GameModeX with VR/mouse is very entertaining.
*HC*_4_	The application creates a better stadium atmosphere using the Oculus Rift VR device.

We decided to follow this confounding-based approach as the *CortexVR* introduces new game modes that differ from the original Helix application which would result in an unequal comparison. Further, the integration of original player paths by the *CortexVR* application creates a different training setting. Additionally, it is also relevant for soccer clubs to know whether VR offers benefits for the training compared to a desktop application before buying VR hardware. Hence, we do not perform a comparison with the original Helix application.

#### 5.2.4. Participants

Thirty-seven participants took part in the study. The age ranged between 21 and 35 (average = 24.89 years; standard deviation = 2.99 years). We had 25 males and 12 female users of which 13 used VR applications before, whereas 24 were first-time users. Sixteen users play regularly video games, 21 not.

#### 5.2.5. Results

**Figure 4:**
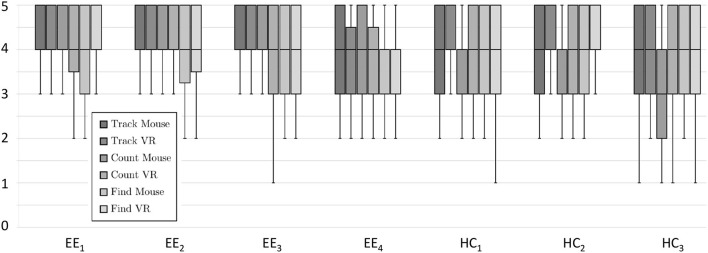
Box-plots of the effort expectancy and the hedonic motivation results (5-points Likert scales). Additionally, the “x” shows the mean value.

The box-plot in Figure 4 visualizes the results of the question items of the user study. The results are grouped by question items. As shown in the box-plot, the results are close to each other for non-VR and VR, especially for Effort Expectancy. Additionally, we did a *t*-test for Effort Expectancy and Hedonic Motivation for analyzing significant effects between the use of the VR and non-VR applications and calculated the effect size using Cohen's d. The results indicate that differences in Effort Efficiency between the non-VR (M = 4.12, SD = 0.24) and the VR version [M = 4.00, SD = 0.19, *t*_(37)_ = 1.31, *p* = 0.2, *d* = 0.5356] are not significant. For Hedonic Motivation, we found that compared to the non-VR version (M = 3.69, SD = 0.20) the user experience is significantly improved with the introduction of VR [M = 3.94, SD = 0.21, *t*_(37)_ = −2.44, *p* = 0.03, *d* = −1.1508].

### 5.3. Adaptive game mode analysis

We applied a quantitative analysis for evaluating the performance of the reinforcement learning algorithm. Using the original implementation of the adaptive tracking game mode bears the risks of having a too steep increase of the difficulty (in case the learner does perform non-optimally) and corrupting the performance of users. Hence, we decided to use data of another study for simulating the use of the tracking game mode. This is possible, as the speed is adjusted only after played rounds, hence, we can use the data to mocking playing a round.

#### 5.3.1. Methodology

We have collected data from 110 different participants, each completed one training session. A training session's data set contains data for each possible level between 13 and $40\frac{km}{h}$. The speed increased by $3\frac{km}{h}$ after three rounds. This results in 30 played rounds per participant. Using this data, we simulated training sessions with the adaptive game mode. We executed one hundred cycles for evaluating the learner. A cycle consisted of one training session with the data from each participant. We feed the learner with the results of a participant for the calculated speed. Then, the learner calculated for each participant the speed of the next level and, again, we used the data for the speed of the participant. We performed thirty rounds (10 levels with three rounds each). Since the reinforcement learning algorithm is updated after each level, we perform around 100, 000 updates.

#### 5.3.2. Results

We analyzed the cumulative reward and the regret of a selected action. The results are described below.

##### 5.3.2.1. Reward

**Figure 5:**
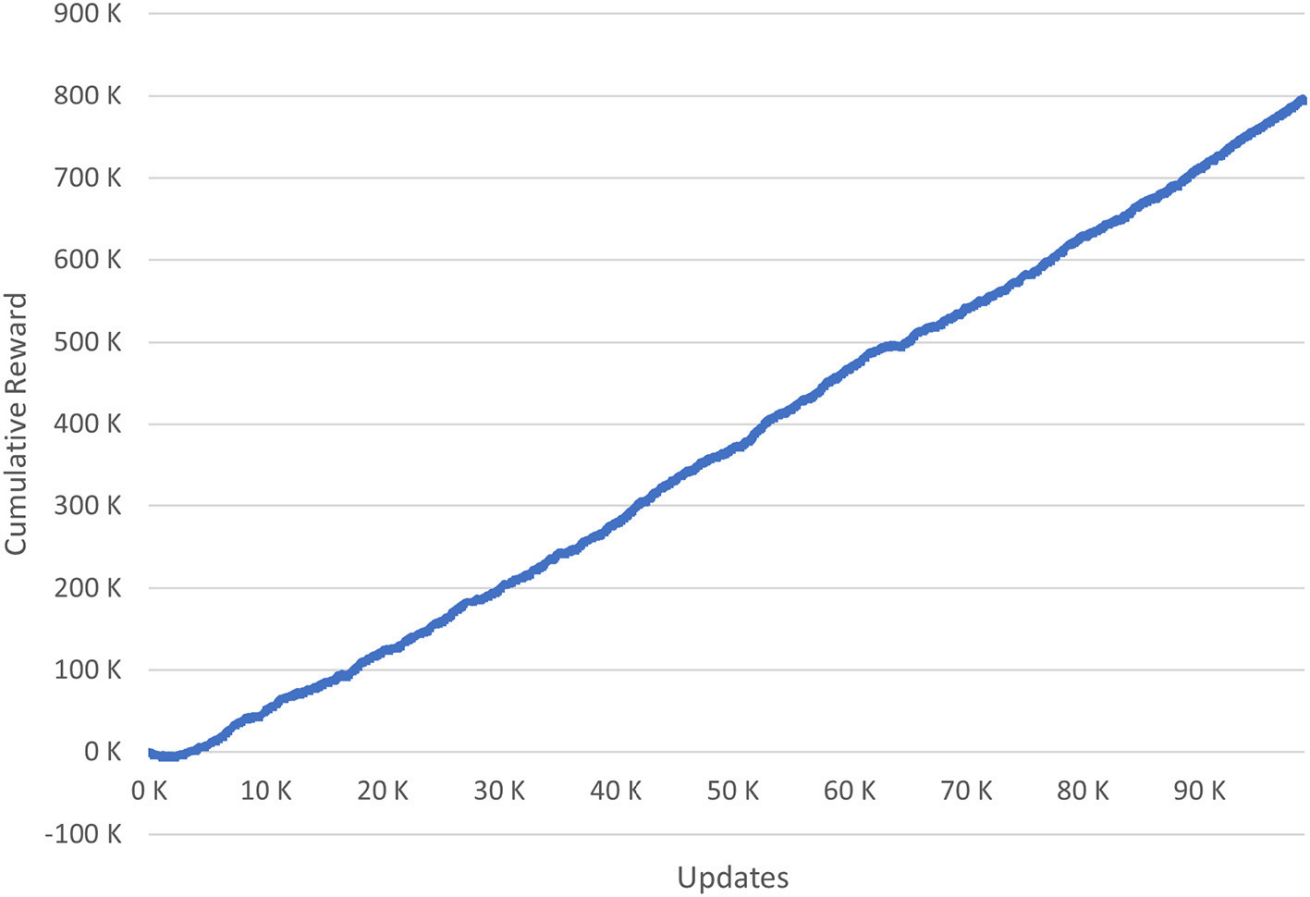
The cumulative reward of the simulation.

First, we consider the reward that the reinforcement learning algorithm is supposed to apply a beneficial policy. It can be negative for actions not useful for achieving the algorithm's goal and positive for expedient ones. The reward is calculated at each update for the executed action. Figure 5 shows the cumulative reward for all updates of the simulation. A mostly linear increase can be recognized. This linear-gradient indicates that the algorithm found a policy not changing significantly. Further, it indicates the policy granting a positive reward on average. Still, the decrease at the beginning of the process needs around 3,300 updates to achieve a break-even point. From this point on, the previously mentioned linear-gradient commences.

**Figure 6:**
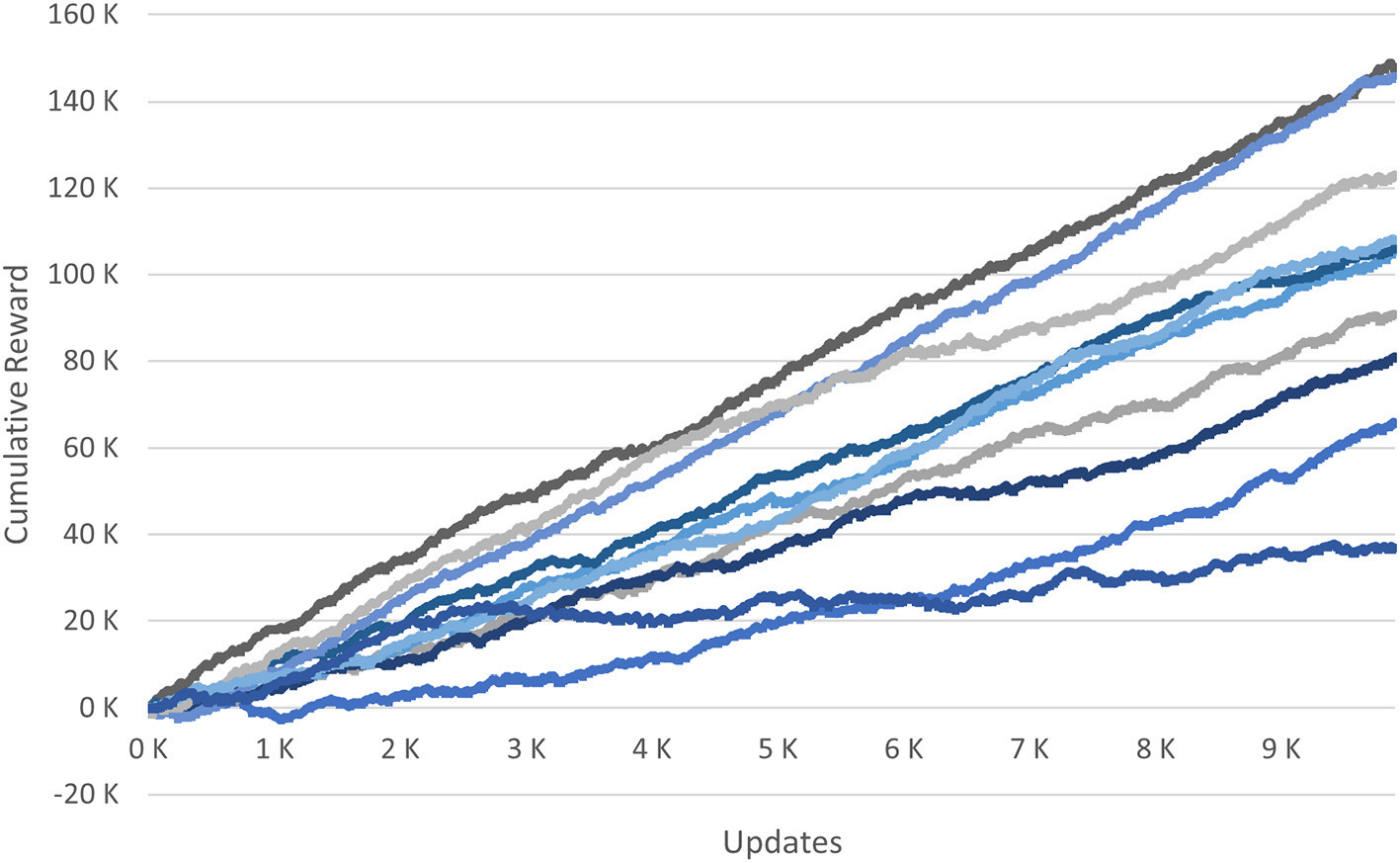
Ten-fold cross-validation for the cumulative reward.

The cross-validation of the reward into ten user groups show that there are clear differences depending on the composition of a group as visible by the gradient of the different functions (see Figure 6). Accordingly, an approach for learning in this use case should cluster the data beforehand into user groups.

##### 5.3.2.2. Regret

**Figure 7:**
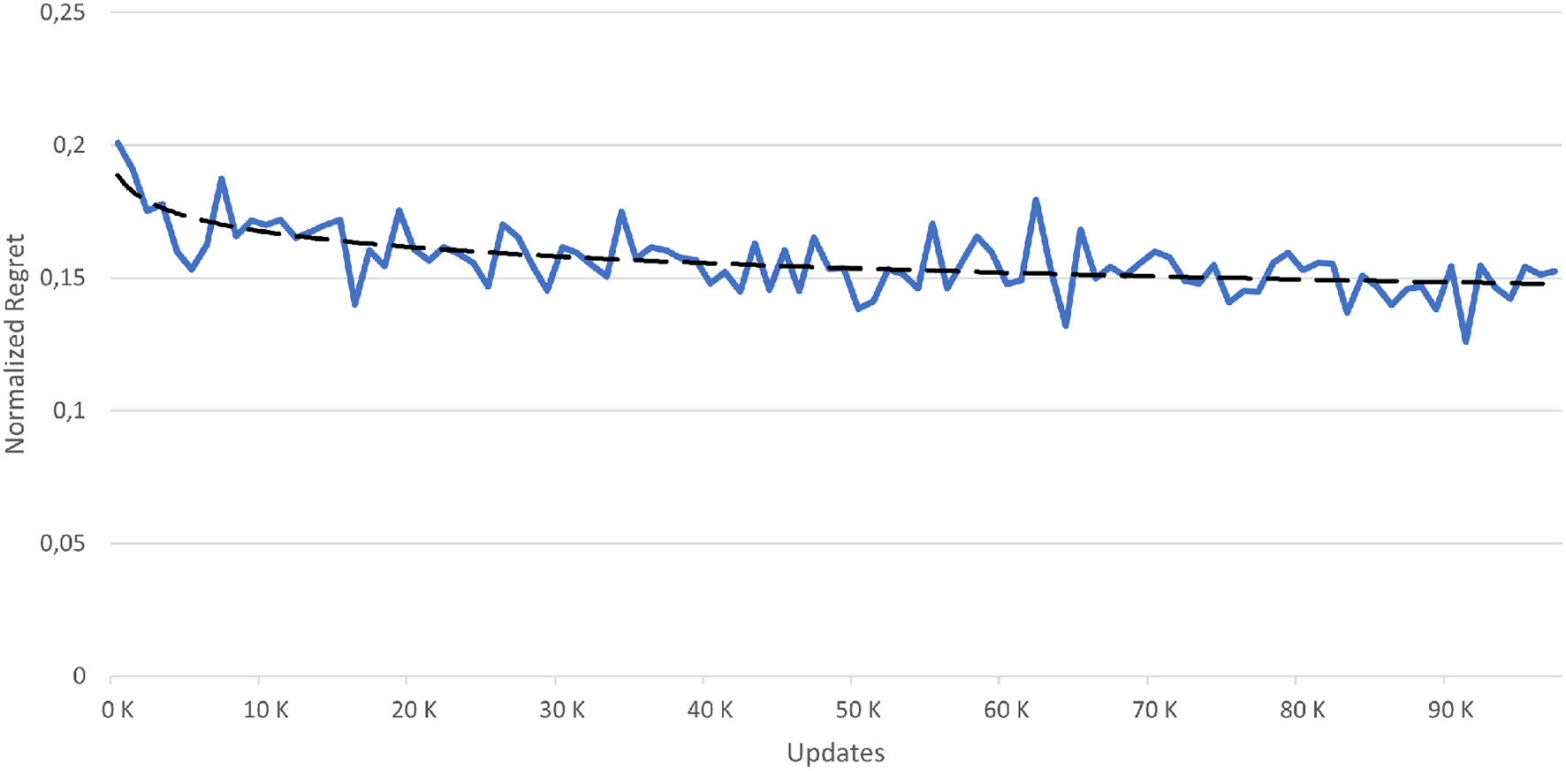
Regret for the reinforcement learning approach.

The regret can be defined as the difference in the reward achieved for a chosen action in comparison to the reward of the best possible action. We define a normalized regret as *normalizedregret* = *regret*(*executedaction*)/*regret*(*worstaction*) whereas the worst action the one with the highest regret is. As a result, the normalized regret stays between 0 (best possible action) and 1 (worst possible action). Figure 7 shows the normalized regret for each update (average for 1,000 steps each) and the development of the score. As one can see, the regret on average decreases with increasing rounds, i.e., with more training. Fluctuations in the regret shows the regularly exploration of new actions.

## 6. Discussion

In this section, we discuss the suitability of the *CortexVR* and *Coach App* applications based on the evaluation. Therefore, this section is divided according to the hypotheses.

In contrast to the former Helix, users of *CortexVR* can directly interact with the application (see requirement *R*_*App*_2), either using mouse/key control or the Oculus Rift VR device (see requirement *R*_*App*_1). As this avoids the need of having a room-filling projection system, the *CortexVR* application is a stand-alone tool (see requirement *R*_*Config*_4) with minimal setup effort. The results show that either mouse/key control or VR is superior in terms of Effort Expectancy (rejecting hypothesis *H1*) depending on the game mode. However, a t-test did not show significance. We could find only weak correlations between the moderators and these results. Some minor correlations exist between the variable of having experience in using VR devices with a satisfaction of the VR control. It is feasible that users with higher experience in using VR applications will show significant better results. Further, training effects might change the results, too. This could be investigated in an extended long-term user study. The metrics shows the superiority of VR regarding Hedonic Motivation (supporting hypothesis *H2*). Especially all participants (fully) agreed that VR creates a better atmosphere. This improves the gamification effects which are a relevant aspect for the effectiveness of the training sessions.

The user study indicates that VR enhances the Hedonic Motivation (cf. hypothesis *H2*). As this might by a result of the novelty of using VR for some participants, we need to investigate the results in more detail within a longitudinal study. We could further investigate whether familiarity with the system improves the handling times with VR-based control. This is an important factor for measuring a user's performance, i.e., supporting the analysis and comparison of players' EFs.

In contrast to the original Helix system, our implementation of the *CortexVR* system supports the analysis of the reaction times of a player in addition to the accuracy. However, the current evaluation focused on comparing the VR-based and the non-VR versions of the application and purposely did not measure efficiency of users in terms of reaction times. These times are highly dependent on the interaction style and input interface, here mouse and 3D controllers, and, especially, those depend on the experience of users with the user interface. However, this would be an analysis of the user interface rather than the efficiency of the system for supporting the users in their task. Still, the new functionality of measuring reaction times enable to use those measures in studies to efficiently compare user as well as observe the training progress of players.

Our system not only supports the analysis of EFs, but can be also used for training of EFs. The most effective method to prove the efficiency of our system in training EFs would be a long-term study of a user group. However, this is not the scope of this paper. Still, a long-term user study for analyzing the effects of training the EFs using our *CortexVR* system is part of our future work, however, this is barely feasible due to the fluctuating nature (due to player transfers or injuries) in the composition of soccer teams.

However, there is a lack of theoretical and empirical justification of the included training tasks. We designed the games modes after discussions with staff and coaches of the soccer clubTSG HoffenheimẆhereas the staff have a lot of experience in this area and also scientific personnel was involved, we did not follow a systematic approach. Open questions include: How can be assured that the games selected improve the proposed EFs? How to set the parameters (number of players to be tracked or counted, number of distractors and degree of similarity with the targets, etc.) of each game in relation to the player's EF level? How to systematically define the complexity of the situations in which the players have to estimate the ball position depending on their EFs?

## 7. Conclusion

This paper presented our approach for training and analyzing EFs, a set of cognitive functions. Different studies have shown that high class soccer players have a dominant level of EFs compared to low class players or non players (Green et al., 2012; Vestberg et al., 2012; Verburgh et al., 2014). In cooperation with the German Bundesligasoccer club TSG Hoffenheim, we designed and implemented the *CortexVR* for VR-based training of the EFs. The *CortexVR* application is complemented by the *Coach App* for configuration of training sessions and analysis of the user performance data.

We evaluated both systems. For the *CortexVR* we designed a user study based on the constructs Effort Expectancy (Venkatesh et al., 2003) and Hedonic Motivation (Venkatesh et al., 2012). Thirty-seven users played the three game modes of the *CortexVR* app with a traditional mouse/key control and using the Oculus Rift VR device with the Oculus controllers. The results support our hypotheses. Regarding the Hedonic Motivation (hypothesis *H2*), the VR-based control is significantly dominating and supports the usage experience. Additionally, the user study showed that depending on the game mode, either the VR-based or the mouse/key-based control is superior in terms of Effort Expectancy (hypothesis *H1*). However, detailed tests in a longitudinal study on the effects for training by using VR are subject to future work.

In this paper, we focused the domain of professional soccer players. However, our approach can be applied for training of EFs in other domains, e.g., for the treatment of Parkinson's disease (Sammer et al., 2006) or children with ADHD (Tamm et al., 2014). Additionally, it might be used for education and training in domains where a fast reaction (in emergency and exceptional situations) is critical, e.g., police, fire brigade, or public transportation. Through the modularity of our system, it can be easily customized and extended, e.g., with new game modes or another game engine. Both measurements of the adaptive game mode, reward, and regret, indicate the correctness of the learner. Still, the benefits of adaptation of the game level have to be analyzed in detail. This was out of scope of this paper but is part of our future work.

## Data availability statement

The raw data supporting the conclusions of this article will be made available by the authors, without undue reservation.

## Ethics statement

Ethical review and approval was not required for the study on human participants in accordance with the local legislation and institutional requirements. The patients/participants provided their written informed consent to participate in this study.

## Author contributions

CKr and JN were initially writing the paper, supporting the user study, and leading the project. JS and ML were responsible for revising the paper and provided advice in the conceptualization of the study and the VR parts. JM, JS, and PE contributed to the conceptualization of the prototype and supported the development with scientific advise regarding the Executive Functions. NB, MB, AH, and CKo were responsible for the development of the CortexVR software. FH was responsible for the development of the adaptive game mode. SK and CB acted as scientific advisor supporting the conceptualization and advising the paper writing process. All authors contributed to the article and approved the submitted version.

## Conflict of interest

The authors declare that the research was conducted in the absence of any commercial or financial relationships that could be construed as a potential conflict of interest.

## Publisher's note

All claims expressed in this article are solely those of the authors and do not necessarily represent those of their affiliated organizations, or those of the publisher, the editors and the reviewers. Any product that may be evaluated in this article, or claim that may be made by its manufacturer, is not guaranteed or endorsed by the publisher.

## Footnotes

^1^A video of the prototype can be found at: https://www.youtube.com/watch?v=gQM2mP5Mjs0.

^2^A video of the original Helix system is available at: https://www.youtube.com/watch?v=M7Tmg1mmgHw&t=11s.

## References

[B1] Akbaş A. Marszałek W. Kamieniarz A. Polechooński Słomka K. J. Juras G. (2019). Application of virtual reality in competitive athletes—a review. J. Hum. Kinet. 69, 5–16. 10.2478/hukin-2019-0023 31666884 PMC6815076

[B2] Baddeley A. (2010). Working memory. Curr. Biol. 20, R136–R140. 10.1016/j.cub.2009.12.014 20178752

[B3] Bejjanki V. R. Zhang R. Li R. Pouget A. Green C. S. Lu Z.-L.. (2014). Action video game play facilitates the development of better perceptual templates. Proc. Natl. Acad. Sci. U.S.A. 111, 16961–16966. 10.1073/pnas.1417056111 25385590 PMC4250112

[B4] Bideau B. Kulpa R. Vignais N. Brault S. Multon F. Craig C. (2010). Using virtual reality to analyze sports performance. IEEE Comput. Graph. Appl. 30, 14–21. 10.1109/MCG.2009.134 20650707

[B5] Bideau B. Multon F. Kulpa R. Fradet L. Arnaldi B. (2004). “Virtual reality applied to sports: Do handball goalkeepers react realistically to simulated synthetic opponents?,” in Proceedings of the 2004 ACM SIGGRAPH International Conference on Virtual Reality Continuum and Its Applications in Industry, 210–216. 10.1145/1044588.1044632

[B6] Bird J. M. (2020). The use of virtual reality head-mounted displays within applied sport psychology. J. Sport Psychol. Action 11, 115–128. 10.1080/21520704.2018.1563573

[B7] Bowman D. A. McMahan R. P. (2007). Virtual reality: how much immersion is enough? Computer 40, 36–43. 10.1109/MC.2007.257

[B8] Büning C. Jürgens L. Lausberg H. (2021). Divergent learning experiences in sports enhance cognitive executive functions and creativity in students. Phys. Educ. Sport Pedag. 26, 402–416. 10.1080/17408989.2020.1812056

[B9] Carling C. (2010). Analysis of physical activity profiles when running with the ball in a professional soccer team. J. Sports Sci. 28, 319–326. 10.1080/02640410903473851 20077273

[B10] Climent-Martinez G. Luna-Lario P. Bombin-Gonzalez I. Cifuentes-Rodriguez A. Tirapu-Ustarroz J. Diaz-Orueta U. (2014). Neuropsychological evaluation of the executive functions by means of virtual reality. Rev. Neurol. 58, 465–475. 10.33588/rn.5810.2013487 24819943

[B11] De Waelle S. Laureys F. Lenoir M. Bennett S. J. Deconinck F. J. (2021). Children involved in team sports show superior executive function compared to their peers involved in self-paced sports. Children 8:264. 10.3390/children8040264 33808250 PMC8065925

[B12] Dye M. W. Green C. S. Bavelier D. (2009). Increasing speed of processing with action video games. Curr. Direct. Psychol. Sci. 18, 321–326. 10.1111/j.1467-8721.2009.01660.x 20485453 PMC2871325

[B13] EonSports VR (2019). EonSports VR Training. Available online at: http://eonsportsvr.com/vr-sports-training (accessed March 25, 2019).

[B14] Gao Z. Lee J. E. McDonough D. J. Albers C. (2020). Virtual reality exercise as a coping strategy for health and wellness promotion in older adults during the COVID-19 pandemic. J. Clin. Med. 9:1986. 10.3390/jcm9061986 32630367 PMC7355577

[B15] Green C. S. Sugarman M. A. Medford K. Klobusicky E. Bavelier D. (2012). The effect of action video game experience on task-switching. Comput. Hum. Behav. 28, 984–994. 10.1016/j.chb.2011.12.020 22393270 PMC3292256

[B16] Harnishfeger K. K. (1995). “The development of cognitive inhibition: theories, definitions, and research evidence,” in Interference and Inhibition in Cognition (Elsevier), 175–204. 10.1016/B978-012208930-5/50007-6

[B17] Holfelder B. Klotzbier T. J. Eisele M. Schott N. (2020). Hot and cool executive function in elite- and amateur- adolescent athletes from open and closed skills sports. Front. Psychol. 11:694. 10.3389/fpsyg.2020.00694 32373029 PMC7177013

[B18] Icaros (2019). Active VR Icaros. Available online at: https://www.icaros.com/ (accessed March 3, 2019).

[B19] Ingle S. (2016). Are We a Step Closer to Being Able to Measure Football IQ? Available online at: https://www.theguardian.com/football/blog/2016/dec/04/barcelona-andres-iniesta-scope-embrace-brain-game-real-madrid (accessed March 25, 2019).

[B20] Koch P. Krenn B. (2021). Executive functions in elite athletes - comparing open-skill and closed-skill sports and considering the role of athletes' past involvement in both sport categories. Psychol. Sport Exerc. 55:101925. 10.1016/j.psychsport.2021.101925

[B21] Kubesch S. Walk L. (2009). Körperliches und kognitives training exekutiver funktionen in kindergarten und schule. Sportwissenschaft 39, 309–317. 10.1007/s12662-009-0079-2

[B22] Lalonde G. Henry M. Drouin-Germain A. Nolin P. Beauchamp M. H. (2013). Assessment of executive function in adolescence: a comparison of traditional and virtual reality tools. J. Neurosci. Methods 219, 76–82. 10.1016/j.jneumeth.2013.07.005 23867080

[B23] Lo Priore C. Castelnuovo G. Liccione D. Liccione D. (2003). Experience with v-store: considerations on presence in virtual environments for effective neuropsychological rehabilitation of executive functions. Cyberpsychol. Behav. 6, 281–287. 10.1089/109493103322011579 12855084

[B24] McGeorge P. Phillips L. H. Crawford J. R. Garden S. E. Sala S. D. Milne A. B.. (2001). Using virtual environments in the assessment of executive dysfunction. Presence 10, 375–383. 10.1162/105474601147023527677827

[B25] McPartland M. Gallagher M. (2011). Reinforcement learning in first person shooter games. IEEE Trans. Comput. Intell. AI Games 3, 43–56. 10.1109/TCIAIG.2010.2100395

[B26] Mi Hiepa Sports (2019). The Global Elite Football VR Platform. Available online at: http://mihiepa.com/ (accessed March 25, 2019).

[B27] Michael Horeni (2017). Innovation—Da Sind Wir Spitze. Available online at: https://www.faz.net/aktuell/sport/fussball/wohin-rollt-der-ball/1899-hoffenheim-maezen-dietmar-hopp-im-interview-ueber-innovation-15128374/helix-auf-der-15131800.html

[B28] NeuroTrainer (2019). Connect Neuroscience to In-Game Performance. Available online at: https://neurotrainer.com/ (accessed March 3, 2019).

[B29] Pugnetti L. Mendozzi L. Attree E. A. Barbieri E. Brooks B. M. Cazzullo C. L.. (1998). Probing memory and executive functions with virtual reality: past and present studies. CyberPsychol. Behav. 1, 151–161. 10.1089/cpb.1998.1.151

[B30] Reaction VR Sports (2019). Virtual Goalie—Virtual Reality Lacrosse Goalie Training. Available online at: https://www.reactionvrsports.com/virtual-goalie-product/ (accessed March 3, 2019).

[B31] Sammer G. Reuter I. Hullmann K. Kaps M. Vaitl D. (2006). Training of executive functions in Parkinson's disease. J. Neurol. Sci. 248, 115–119. 10.1016/j.jns.2006.05.028 16765378

[B32] Scott W. A. (1962). Cognitive complexity and cognitive flexibility. Sociometry 25, 405–414. 10.2307/2785779

[B33] Strauss E. Sherman E. M. Spreen O. (2006). A Compendium of Neuropsychological Tests: Administration, Norms, and Commentary. American Chemical Society.

[B34] StriVR (2019). StriVR Sports. Available online at: https://www.strivr.com/sports/ (accessed March 3, 2019).

[B35] Sturtevant N. R. White A. M. (2007). “Feature construction for reinforcement learning in hearts,” in Computers and Games, Vol. 4630 of Lecture Notes in Computer Science, eds D. Hutchison, T. Kanade, J. Kittler, J. M. Kleinberg, F. Mattern, C. Mitchell, M. Naor, O. Nierstrasz, C. Pandu Rangan, B. Steffen, M. Sudan, D. Terzopoulos, D. Tygar, M. Y. Vardi, G. Weikum, H. J. van den Herik, P. Ciancarini, and H. H. L. M. Donkers (Berlin; Heidelberg: Springer Berlin Heidelberg), 122–134. 10.1007/978-3-540-75538-8_11

[B36] Success Series (2019). Success Series. Available online at: http://www.successseries.com/ (accessed March 3, 2019).

[B37] Tamm L. Nakonezny P. A. Hughes C. W. (2014). An open trial of a metacognitive executive function training for young children with ADHD. J. Attent. Disord. 18, 551–559. 10.1177/1087054712445782 22647287

[B38] Tesauro G. (1995). Temporal difference learning and td-gammon. Commun. ACM 38, 58–68. 10.1145/203330.203343

[B39] Venkatesh V. Morris M. Davis G. Davis F. (2003). User acceptance of information technology: toward a unified view. MIS Q. 27, 425–478. 10.2307/30036540

[B40] Venkatesh V. Thong J. Y. L. Xu X. (2012). Consumer acceptance and use of information technology: extending the unified theory of acceptance and use of technology. MIS Q. 36, 157–178. 10.2307/41410412

[B41] Verburgh L. Scherder E. J. A. van Lange P. A. Oosterlaan J. (2014). Executive functioning in highly talented soccer players. PLoS ONE 9:e91254. 10.1371/journal.pone.0091254 24632735 PMC3954684

[B42] Vestberg T. Gustafson R. Maurex L. Ingvar M. Petrovic P. (2012). Executive functions predict the success of top-soccer players. PLoS ONE 7:e34731. 10.1371/journal.pone.0034731 22496850 PMC3319604

[B43] Weiss P. L. Kizony R. Feintuch U. Katz N. (2006). Virtual reality in neurorehabilitation. Textb Neural Repair Rehabil. 51, 182–197. 10.1017/CBO9780511545078.015

